# Inhibition of Lysine-Specific Demethylase-1 (LSD1/KDM1A) Promotes the Adipogenic Differentiation of hESCs Through H3K4 Methylation

**DOI:** 10.1007/s12015-016-9650-z

**Published:** 2016-04-08

**Authors:** Yujing Xiong, Enyin Wang, Yan Huang, Xiaoyi Guo, Yiping Yu, Qingyun Du, Xiaoyan Ding, Yingpu Sun

**Affiliations:** Reproductive Medical Centre, The First Affiliated Hospital of Zhengzhou University, 1 Jianshe East Road, Zhengzhou, 450052 Henan People’s Republic of China; Institute of Biochemistry and Cell Biology, Shanghai Institutes for Biological Sciences, Chinese Academy of Sciences, No. 320 Yueyang Road, Shanghai, 200031 People’s Republic of China

**Keywords:** LSD1, hESCs, Adipogenesis, CBB1007, Histone modification

## Abstract

Given their totipotency, human embryonic stem cells (hESCs) can differentiate into all types of cells, including adipocytes, and provide an excellent research model for studying diseases associated with the metabolism of adipocytes, such as obesity and diabetes mellitus. Epigenetic regulation, including DNA methylation and histone modification, plays an essential role in the development and differentiation of hESCs. Lysine-specific demethylase 1 (LSD1), a well-characterized histone-modifying enzyme, demethylates dimethylated histone H3 lysine 4 (H3K4) through a flavin adenine dinucleotide (FAD)-dependent oxidative reaction. LSD1 affects the growth and differentiation of human and mouse ES cells, and the deletion of this gene in mice leads to embryonic lethality. Here, we investigated the functional role of LSD1 during the adipogenic differentiation of hESCs involving the demethylation of H3K4. We also found that treating hESCs with the LSD1 inhibitor CBB1007 promotes the adipogenic differentiation of hESCs.

## Introduction

In 1998, human embryonic stem cells (hESCs) were first isolated by Thomson et al. [1] and have become a potential source for cell replacement therapies because of their abilities to self-renew and maintain pluripotency with the potential to differentiate into all types of cells. The epigenetic modification of chromosomes plays an important role in ES cell maintenance and differentiation [2]. An important aspect of epigenetic regulation is posttranslational histone tail modification, including acetylation, methylation, citrullination, phosphorylation, ubiquitylation, sumoylation, and biotinylation [3]. Among these modifications, methylation marks are dynamically regulated by histone methyltransferases and demethylases [4]. Several studies have reported the role of the dynamic regulation of histone tail methylation in the maintenance of pluripotency in stem cells to block cell differentiation [5, 6]. Histone demethylases have also been reported to be associated with a broad spectrum of developmental functions in gametogenesis, embryogenesis and differentiation [7, 8].

Lysine-specific demethylase 1 (LSD1/KDM1A) [9] is a flavin-containing amino oxidase (AO) that mainly demethylates dimethylated histone H3 lysine 4 (H3K4) through a flavin adenine dinucleotide (FAD)-dependent oxidative reaction but does not alter trimethylated H3K4 [10, 11]. Although LSD1 is important for maintaining the pluripotency of ES cells [12–14] and the knockdown of LSD1 results in decreased differentiation of lineage-committed 3 T3-L1 preadipocytes into adipocytes [15], the role of LSD1 in inducing adipogenesis from hESCs has not been explored. Various LSD1 inhibitors have been discovered, and their inhibitory efficiencies have been reported [16, 17]. CBB1007 is one of several competitive LSD1 inhibitors that were developed based on the structure of LSD1 with a peptide inhibitor derived from the N-terminal tail of histone H3 [18] that can efficiently inhibit the LSD1-mediated demethylation of H3K4me2 [19]. Here, we explored the role and mechanism of LSD1 in induced adipogenesis of hESCs using the LSD1 inhibitor CBB1007 and illuminated the role of epigenetic regulation in adipogenesis.

## Materials and Methods

### Cell Culture

The classical human embryonic stem cell line H9 was kindly provided by the National Stem Cell Bank c/o WiCell Research Institute [1] and was routinely propagated on a feeder layer composed of inactivated mouse embryonic fibroblast (MEF) [20]. The cultures were maintained in an incubator at 37 °C with a humidified atmosphere of 5 % CO_2_ in air. MEFs separated from ICR fetal mice at gestation day 12.5 were purchased from Beijing Weitong Lihua Experimental Animal Technology Co. Ltd. The hESCs were incubated on an inactivated feeder layer. Primary MEFs from passages 1 to 4 were inactivated with mitomycin C to prepare the feeder layer. One passage of hESCs was performed using the mechanical method every 5 to 7 days. The hESC culture medium contained 80 % Dulbecco’s modified Eagle medium (DMEM/F12 (Invitrogen; 11330–032), 20 % knockout serum replacement (Invitrogen; 10828–028), 2 mM GlutaMAX (Invitrogen; 25030–081), 1 % nonessential amino acids (NEAAs) (Invitrogen; 11140), 0.25 % 55 mM 2-mercaptoethanol (Invitrogen; 21985), and 4 ng/mL basic fibroblast growth factor (bFGF) (Invitrogen; 13256).

### Adipogenic Differentiation and LSD1 Inhibitor Treatment

H9 cells were differentiated into adipocytes directly by changing the normal culture medium to an adipogenic differentiation cocktail, which consisted of 90 % α–MEM, 10 % knockout serum replacement, 10 μg/mL insulin, 0.5 mmol/L 3-isobutyl-1-methylxanthine (IBMX), 10 μmol/L rosiglitazone and 1 μmol/L dexamethasone. The adipogenic induction culture lasted for 14 days with daily medium changes, and the cell colonies were then observed under an inverted microscope. CBB1007 (Millipore, Billerica, MA, USA) at concentrations of 5 mM,10 mM and 20 mM was added to the adipogenic induction medium as the experimental groups from day 1 to day 14 of induction. In contrast, CBB1007 was not added to the control group.

### Oil Red O Staining

After the removal of the medium, differentiated hESCs on day 14 were washed twice with PBS, fixed with 10 % paraformaldehyde for 30 min, and then stained with diluted Oil Red O for 10 min at room temperature. The cells were washed with 75 % alcohol to remove the unbound dyes and stained with hematoxylin for 1 min. After washing with PBS, the cells were observed under an inverted microscope.

### RNA Extraction and Real-Time Polymerase Chain Reaction (RT-PCR)

The total cellular RNA from undifferentiated hESCs cultured in proliferation medium and from hESCs cultured in differentiation medium for 14 days was extracted using the RNeasy plus mini kit (Qiagen 74134) according to the suggestions provided by the supplier. For real-time polymerase chain reaction (RT-PCR), cDNA was synthesized using iScript (BIORAD). RT-PCR was performed on an Applied Biosystems 7500 Real-Time PCR System equipped with a 96-well reaction plate. Relative quantification of the mRNA levels was performed using the comparative cycle threshold (Ct) method using β-actin as the reference gene. The primers used for RT-PCR are listed in Table 1.

**Table 1:** Target gene primers used in the RT-qPCR analysis

Target gene	Primer sequences
PRARγ-2 forward	5’-GACAAAATATCAGTGTGAATTACAGC-3’
PRARγ-2 reverse	5’-CCCAATAGCCGTATCTGGAAGG-3’
C/EBPα forward	5’-AGAAGGCTGGGGCTCATTTG-3’
C/EBPα reverse	5’-AGGGGCCATCCACAGTCTTC-3’
LSD1 forward	5’-ACCCGCTCCACGAGTCAAAC-3’
LSD1 reverse	5’-ACGCCAACGAGACACCACAG-3’
β-actin forward	5’-CTCCATCCTGGCCTCGCTGT-3’
β-actin reverse	5’-GCTGTCACCTTCACCGTTCC-3’

### Protein Extraction and Western Blotting Detection

After washing twice with PBS, the undifferentiated and differentiated cells were harvested by the addition of ice-cold cell lysis buffer (Cell Signaling; #9803) and a protease inhibitor-phenylmethanesulfonyl fluoride (PMSF) (Cell Signaling; #8553) mixture into the culture system. Fifty micrograms of protein extracts were resolved by SDS-polyacrylamide gel electrophoresis (SDS-PAGE) and electrotransferred onto polyvinylidene difluoride membranes. The membranes were blocked for 2 h at room temperature with 5 % nonfat dry milk in Tris-buffered saline and then stored in a box with the primary antibodies, which were diluted in 5 % nonfat milk/Tris-buffered saline, overnight with shaking at 4 °C. Subsequently, the membranes were washed thrice with TBS-T and then incubated with a horseradish peroxidase-conjugated secondary antibody with shaking for 1.5 h at 37 °C. The probed membranes were then incubated in 2 mL of ECL detection reagent for 4 min and subjected to image detection in a darkroom. GAPDH served as the control. The film was scanned according to the manufacturer’s suggestion, and a gel imaging system and an image analysis software were used to analyze the results. Anti-histone H3 peptide with dimethylated lysine 4 (H3K4me2; ab32356), anti-histone H3 (ab61251) and LSD1 (ab129195) antibodies were from Abcam.

### Statistical Analysis

Two-tailed Student’s *t*-test was used to compare the average values between two groups, and analysis of variance (ANOVA) was conducted for multiple comparisons. These tests were performed using SPSS version 20.0. All of the values are shown as the means ± standard deviations (SDs). P-values less than 0.05 were considered to be statistically significant.

## Results

### Adipogenic Differentiation of hESCs in Vitro

**Fig. 1:**
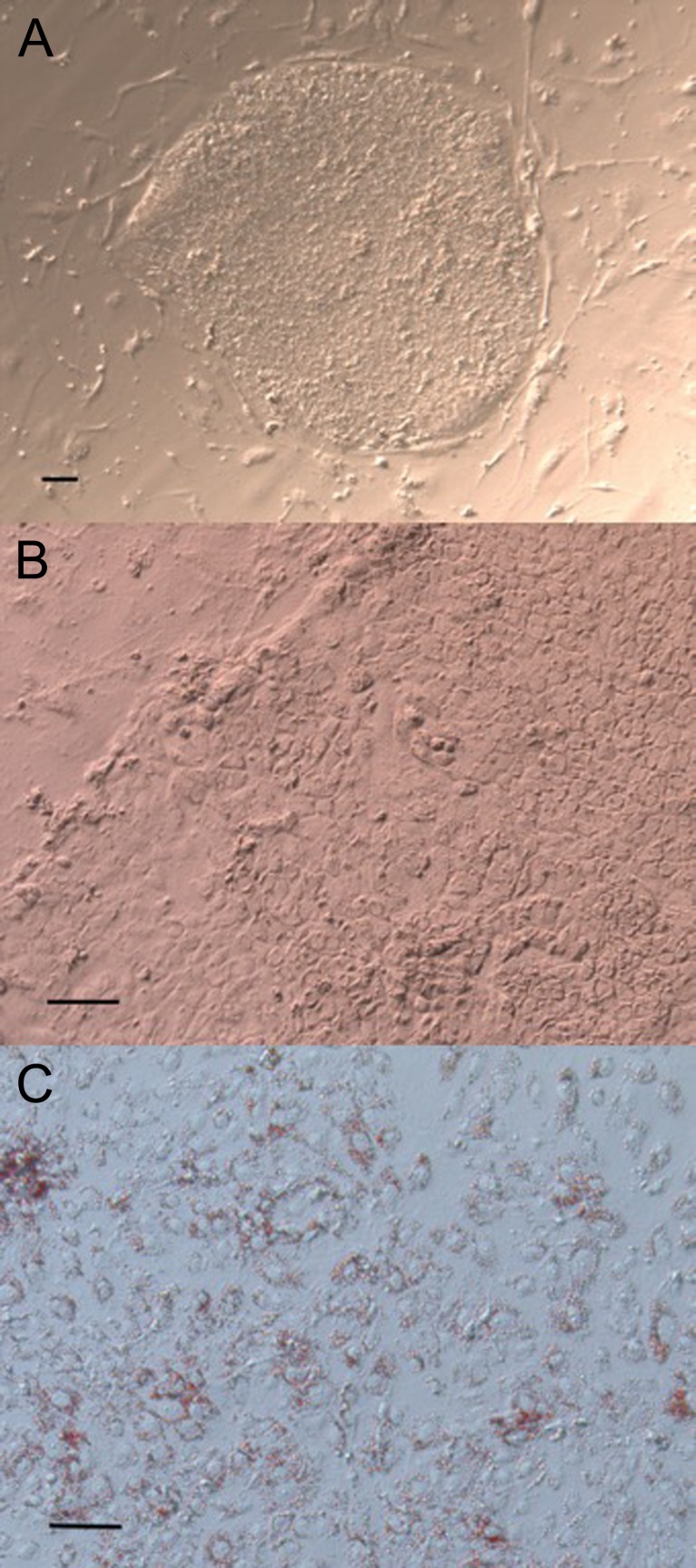
Adipogenic differentiation of hESCs in vitro. **a**. Undifferentiated status of H9 colonies that were chosen to undergo adipogenic differentiation. The colonies appeared flat and elliptical in shape and exhibited clear, smooth cell boundaries. **b**. The morphology of the colonies after day 14 of induction. Only one layer of cells remained. The shape of the cells changed from round to irregular. Additionally, the colonies extended out to become larger. **c**. Oil Red O staining on day 14 after induction. The lipid droplets were stained with red and showed that the adipogenesis was successful. Scale bar = 20 μm

The human embryonic stem cell line H9 was differentiated into adipocytes by directly adding adipogenic medium cocktail into the culture system for 14 days. Before induction, we chose H9 colonies with an undifferentiated status that had flat, elliptically shaped and clear, smooth cell boundaries (Fig. 1a). After 1 day of induction, the shape and boundaries of the colonies changed to have an irregular edge, signs of apoptosis were noted in cells on the top of the colonies and the colonies became loose. On subsequent days, the colonies became looser, and more cells underwent apoptosis mainly on the top and near the edge. The shape of the cells also clearly changed. On day 14 of induction, the colonies only had one layer of cells remaining. The morphology of the cells changed from round to irregular, and the colonies became larger (Fig. 1b). And then the Oil Red O staining was used to identify that the adipogenesis was successful (Fig. 1c).

After 7 days of induction, the cell colonies exhibited lipid accumulation, as determined by Oil Red O staining, although the lipid droplets in the different groups appeared to have different densities. However, after 14 days of induction, more prominent lipid droplets were shown by Oil Red O staining under the microscope. Additionally, the expression of a set of adipocyte markers, including peroxisome proliferator-activated receptor γ-2 (PPARγ-2) and CCAAT/enhancer-binding protein (C/EBP) α, was detected through RT-qPCR analysis. We found that PPARγ-2 and C/EBPα were expressed in differentiated cells but not in undifferentiated cells, demonstrating that adipogenic differentiation from hESCs was successful.

### The LSD1 Inhibitor CBB1007 Promotes the Adipogenic Differentiation of hESCs

**Fig. 2:**
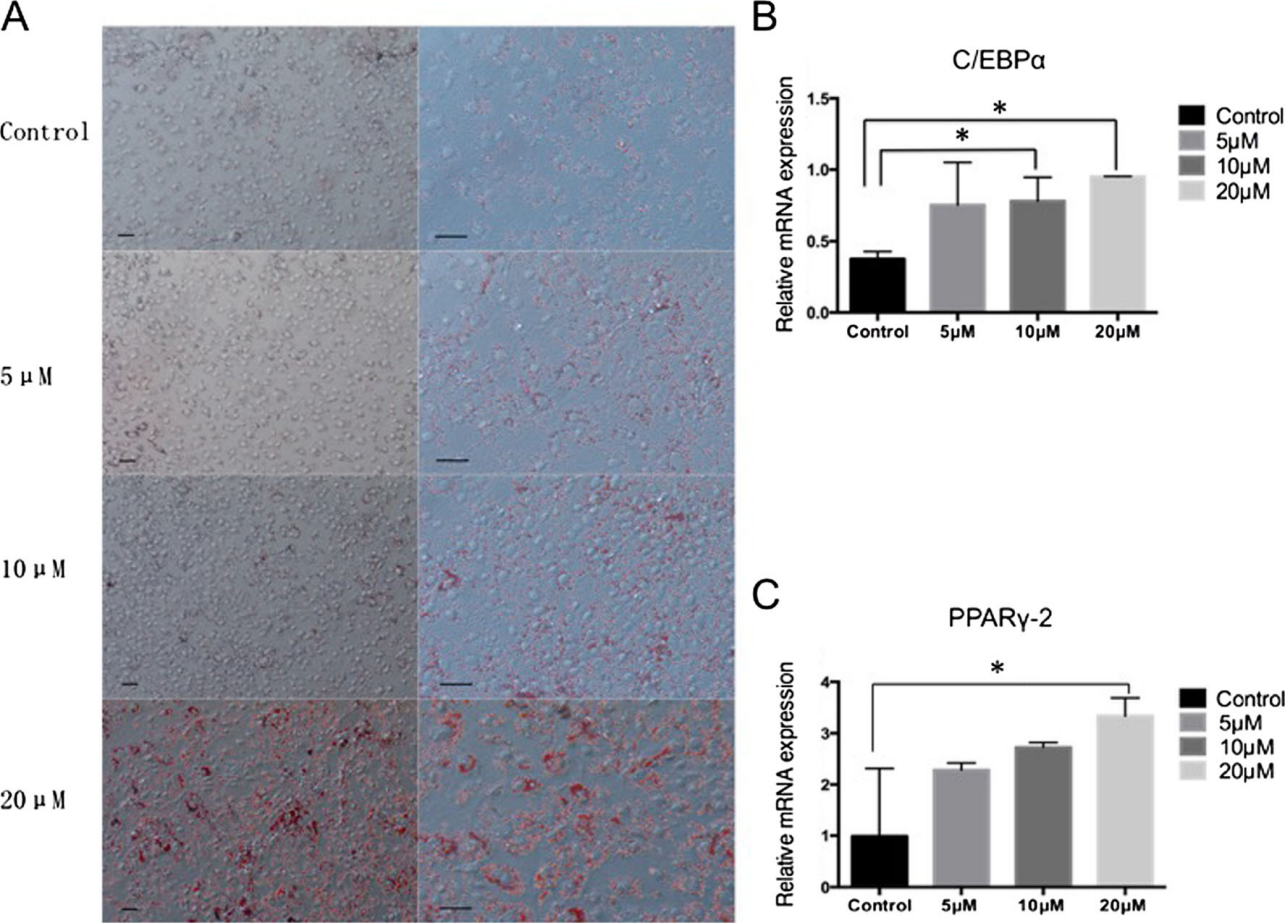
The LSD1 inhibitor CBB1007 promotes the adipogenic differentiation of hESCs. **a**. Oil Red O staining on day 14 after induction. After 14 days of induction, as the dose of CBB1007 increased, the density of the lipid droplets increased. The color of the lipid droplets was red, and some of the droplets fused together due to the high density. Scale bar = 20 μm. **b**. C. qRT-PCR analysis of the relative gene expression of a set of adipocyte markers, PPARγ-2 and C/EBPα, on day 14 after induction. The *bars* indicate the standard error of the mean (SEM) of the two genes. The results of the experiments were independently confirmed three times. The *error bars* represent SEMs for duplicate samples. The significant differences were analyzed by one-way ANOVA. ∗, *P* < 0.05

According to the dose of the LSD1 inhibitor CBB1007 that was added to the induction medium cocktail, the cultures were divided into 5 groups: control group, 5-μM group, 10-μM group, and 20-μM group. After 14 days of induction, all of the groups were processed by Oil Red O staining to detect adipocyte accumulation. Additionally, as the dose of CBB1007 increased, the density of the lipid droplets increased. The color of the lipid droplets was red, and some of the droplets fused together due to their high density (Fig. 2a).

Additionally, the RT-qPCR results indicated that PPARγ-2 and C/EBPα expression increased with increasing doses of CBB1007 (Fig. 2b-c). By comparing the Oil Red O staining and RT-qPCR results between the groups, we noted that the efficiency of the adipogenic differentiation of hESCs increased as the dose of the LSD1 inhibitor CBB1007 increased.

### The LSD1 Inhibitor CBB1007 Increased H3K4me2 Levels

**Fig. 3:**
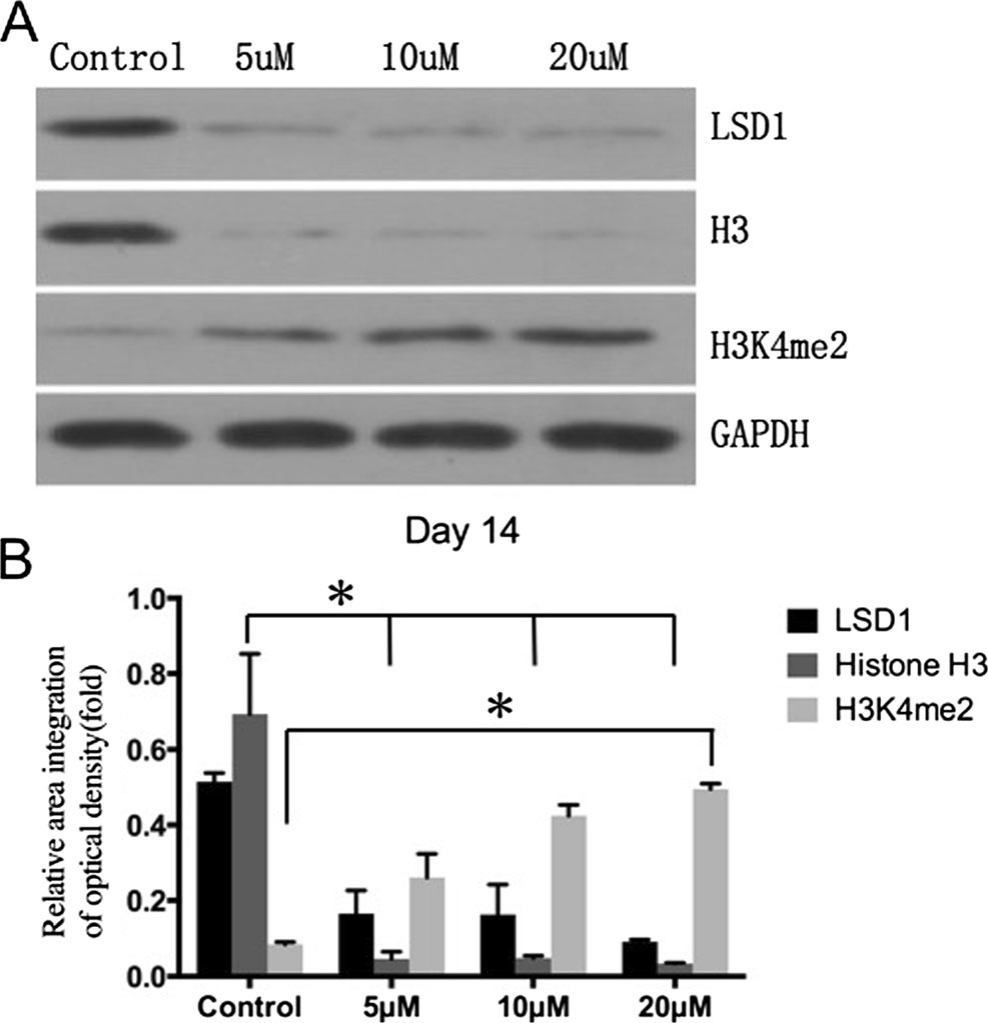
The LSD1 inhibitor CBB1007 increased H3K4me2 levels. **a**. The expression levels of LSD1, histone H3, and H3K4me2 on day 14 after induction were detected by Western blotting. In the control group to the 20-μM group, the expression of LSD1 and histone H3 decreased, whereas the level of histone H3K4 dimethylation (H3K4me2) increased with the increased dose of CBB1007. GAPDH was used as the internal standard. **b**. Densitometry analysis of the bands from the phosphorylated forms of LSD1, histone H3 and H3K4me2. With increasing CBB1007 dose, LSD1 and histone H3 decreased together on day 14. However, based on the demethylation role of LSD1, H3K4me2 increased and exhibited an opposite expression tendency compared with LSD1. The results of the experiments were independently confirmed thrice. The *error bars* represent SEMs for duplicate samples. The significant differences were analyzed by one-way ANOVA.∗, *P* < 0.05

To investigate the role of LSD1 in the induced differentiation of adipocytes, a western blotting analysis was performed to detect the expression of LSD1, histone H3 and H3K4me2 in every group on day 14 of differentiation (Fig. 3a). After 14 days of induction, the expression levels of LSD1 and histone H3 decreased from the control group to the 20-μM group, whereas the level of histone H3K4 dimethylation (H3K4me2) increased with increasing doses of CBB1007. GAPDH was used as the internal standard.

Additionally, densitometry analysis of the bands from the phosphorylated forms of LSD1, histone H3 and H3K4me2 was performed to analyze the relative expression level. With increasing CBB1007 doses, the levels of LSD1 and histone H3 maintained the same expression tendency—i.e., LSD1 and histone H3 decreased together on day 14. However, because of the demethylation role of LSD1, H3K4me2 exhibited the opposite tendency compared with LSD1 (Fig. 3b).

## Discussion

This study is the first to study the role of LSD1 in the induction of adipogenic differentiation in hESCs. By adding CBB1007 during differentiation, we found that inhibition of the lysine specific demethylase-1 LSD1 promotes adipogenic differentiation from hESCs through H3K4 methylation.

Here, we used an adipogenic cocktail that included insulin, dexamethasone, 3-isobutyl-1-methylxanthine (IBMX) and rosiglitazone to induce the differentiation of hESCs into adipocytes. Insulin is the most important part of the cocktail. Insulin plays an important role in glucose transport, glycemic control and glycogen synthesis. Insulin helps adipocytes transport glucose by binding to insulin receptors on the surface of the adipocyte membrane. Thus, insulin enhanced the activation of lipoprotein lipase and promoted the synthesis of adipocytes. Additionally, insulin at concentrations higher than physiological doses can improve preadipocyte proliferation by acting on insulin-like growth factor-1 [21].

Dexamethasone induces the expression of C/EBPα and PPARγ and activates these proteins by increasing the expression of EEAAT/enhancer-binding protein δ (C/EBP δ) and forming a complex with C/EBP δ, resulting in the acceleration of the efficiency of adipocyte differentiation [22]. IBMX represses the activation of cyclic adenosine monophosphate (cAMP) phosphodiesterase and increases the activation of adenylate cyclase, resulting in increased cAMP expression. Additionally, cAMP reacts with cAMP response elements in the cell nucleus by activating cAMP response element binding protein, thus leading to induced expression of adipogenic genes, such as C/EBPβ [23]. Rosiglitazone, a member of the thiazolidinedione (TZD) class of medicinal drugs, can highly selectively activate PPARγ receptors, resulting in highly efficient adipocyte synthesis and retention. In the early phase of preadipocyte differentiation, adipogenic stimuli induce low-level PPARγ and C/EBPα expression. Subsequently, these proteins promote each other’s expression to form a positive feedback loop. The resulting high levels of PPARγ and C/EBPα activate adipocyte gene expression and thereby lipid accumulation, ultimately leading to the phenotypic conversion of hESCs to adipocytes [24].

LSD1 is a novel regulator of lipid metabolism [25]. In our study, we first hypothesized that LSD1 promotes or inhibits the induced differentiation of hESCs because it has been reported that its action is irreplaceable at phenotypic switch points at multiple stages of stem cell development, such as adipogenesis [26]. High expression levels of LSD1 are critical for maintaining hESCs in an undifferentiated status and lead to embryonic lethality in knockout mice [13, 27]. The knockdown of LSD1 in a mouse cell culture model also prevented the epithelial-to-mesenchymal transition [28]. However, we demonstrated that the inhibitor LSD1 promotes adipocyte differentiation from hESCs.

In our study, CBB1007, an inhibitor of LSD1, was used to assess the role of LSD1 in the adipogenic differentiation from hESCs. Next, we demonstrated that the amidino-guanidinium compound that acts as a reversible and substrate competitive LSD1-selective inhibitor [18] could dramatically suppress the expression of LSD1 but enhance H3K4 methylation, thus effectively promoting the adipogenic differentiation of hESCs.

In general, LSD1 is enriched at core promoter regions and enhancers of embryonic stem cells, as detected by chromatin immunoprecipitation (ChIP)-sequencing analysis [14, 29]. LSD1 acts as a transcriptional repressor and is a component of various transcriptional corepressor complexes [5, 30–33], whereas histone H3K4 methylation is generally associated with gene activation and the upregulation of transcription through the recruitment of nucleosome remodeling enzymes and histone acetylates [34]. H3K4 dimethylation is observed on the transcription factors PPARγ and C/EBPα, both of which are the master regulators of adipogenesis [15]. The latter finding indicates that the inhibition of histone demethylase LSD1 increases histone H3K4 dimethylation (H3K4me2) on the PPARγ and C/EBPα promoters, facilitating the expression of PPARγ and C/EBPα and leading to increased adipogenesis.

In this regard, we provide the first demonstration of an association between the H3K4 histone demethylase LSD1 and the induced differentiation of adipocytes from hESCs. In the control group, the expression of LSD1 increased on day 14 of induction compared with day 0. However, after inhibition by CBB1007, the expression of LSD1 was sharply repressed. As a result, the efficiency of adipogenesis increased as shown by Oil Red O staining and PPARγ and C/EBPα expression, which were positively correlated with the H3K4 dimethylation.

The induced differentiation of adipocytes from hESCs provides an excellent model system to study the role of histone demethylase LSD1 in the regulation of stem cell differentiation. Based on our results, we conclude that the LSD1inhibitor CBB1007 promotes the induced differentiation of adipocytes from hESCs, and LSD1 may repress the gene expression associated with adipogenic differentiation by H3K4 demethylation.

In conclusion, our study demonstrates that the LSD1 inhibitor CBB1007 promotes the adipogenic differentiation of hESCs through H3K4 methylation. Our work also sheds light on the functional role and molecular mechanism of the histone H3K4 demethylase LSD1 in the adipogenic differentiation of hESCs.
